# IUC26657-91 Sex-Based Disparities in Metastatic Renal Cell Carcinoma: Real-World Data from Meet-URO33 Study

**DOI:** 10.1093/oncolo/oyag286.016

**Published:** 2026-08-21

**Authors:** M Zinelli Ronzoni, M Maffezzoli, E Lai, E Verzoni, C Lolli, M Di Napoli, A Signori, S Buti, S E Rebuzzi, D Bimbatti

**Affiliations:** Medical Oncology Unit, University Hospital of Parma; Medicine and Surgery Department, University of Parma, Parma - Italy; Medical Oncology Unit, University Hospital of Parma; Medicine and Surgery Department, University of Parma, Parma - Italy; Oncology Unit 3, Veneto Institute of Oncology IOV-IRCCS, Padua - Italy; Genitourinary Oncology Unit, Medical Oncology Department, Fondazione IRCCS Istituto Nazionale dei Tumori, Milan - Italy; Department of Medical Oncology, IRCCS Istituto Romagnolo per lo Studio dei Tumori (IRST) “Dino Amadori”, Meldola - Italy; Department of Urology and Gynecology, Istituto Nazionale Tumori IRCCS Fondazione G. Pascale, Naples - Italy; Department of Health Sciences, Section of Biostatistics, University of Genova, Genoa; IRCCS Azienda Ospedaliera Metropolitana, Genova - Italy; Medical Oncology Unit, University Hospital of Parma; Medicine and Surgery Department, University of Parma, Parma - Italy; Medical Oncology Unit 2, Ospedale Molinette, Azienda Ospedaliero-Universitaria Città della Salute e della Scienza di Torino, Torino - Italy; Oncology Unit 3, Veneto Institute of Oncology IOV-IRCCS, Padua - Italy

## Abstract

**Background:**

Sex-related biological differences may affect cancer immunity and outcomes with immune checkpoint inhibitors (ICIs) in patients with metastatic renal cell carcinoma (mRCC). This sub-analysis aimed to explore sex-based differences in a real-world cohort of patients with mRCC treated with first-line systemic therapy within the Meet-URO 33 (REGAL) study.

**Methods:**

The Meet-URO 33 study is a multicentre ambispective registry collecting real-world data on patients with mRCC. Clinical-pathological features, haematological parameters, toxicities, and outcomes were compared by sex and age (< 50 vs ≥ 50 years).

**Results:**

Among 1560 patients, 401 were females, and 1159 were males. Females presented with lower BMI (≤ 25 kg/m^2^, 52% *vs* 43%, *p*  =  0.008) and lower median NLR (2.8 vs 3.1, *p*  =  0.005), and had worse baseline characteristics, such as liver metastases (18% *vs* 11%, *p*  =  0.002) and sarcomatoid variant (20.3% *vs* 14.5%, *p*  =  0.025). Grade 3–4 adverse events (AEs) were more frequent in females (35% *vs* 28%, *p*  =  0.012), but no sex-related differences emerged in the pattern of AEs and discontinuation rates. No significant differences were observed in PFS (14.8 *vs* 17.6 months) and OS (39.3 *vs* 39.7 months). Females < 50 years experienced a shorter PFS (8.5 *vs* 19.6 months, HR 2.02, *p*  =  0.009), Fig 1. In multivariable Cox complete-case analyses, sex was not independently associated with either PFS (HR 1.21, 95% CI: 0.97–1.51, p  =  0.099) or OS (HR 1.16, 95% CI: 0.87–1.55, p  =  0.307), and sensitivity analysis based on multiple imputation produced consistent results.

**Conclusions:**

Female patients with mRCC presented with more adverse baseline features and experienced higher severe toxicity rates, although PFS and OS were comparable to male patients. However, multivariable analyses demonstrate that sex does not have an independent prognostic value. Subgroup analysis in female patients aged < 50 years showed worse PFS, suggesting a potential hormonal influence warranting further investigation. Stronger female representation and translational research are needed to guide sex-tailored strategies in advanced RCC.

**Acknowledgements:**

The authors thank Meet-URO33 centres and investigators.

**IUC26657-91 Figure 1:**
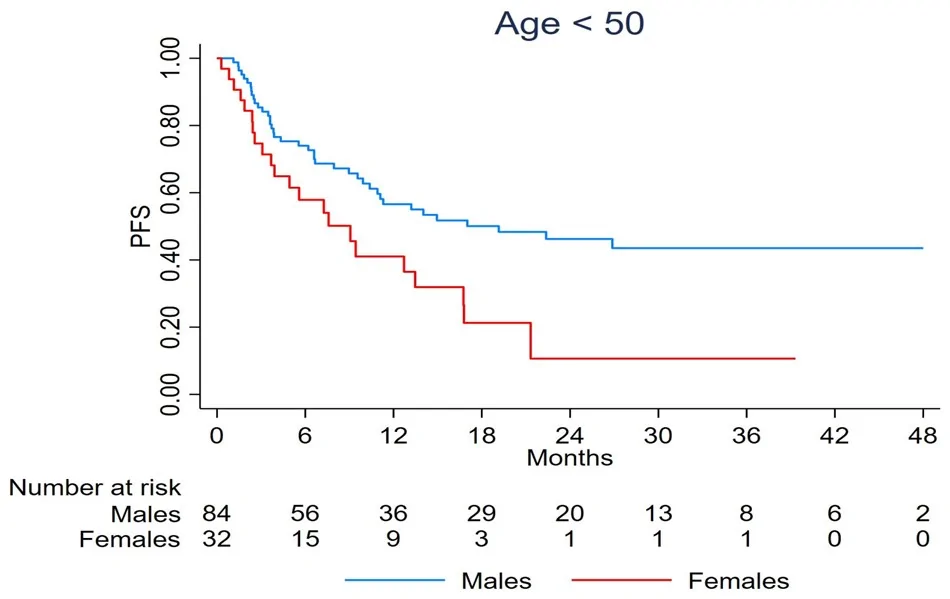
Progression-free survival for first-line treatment in female and male patients aged < 50 years.

